# Opa1 and MT-Nd6 mutations induce early mitochondrial changes in the retina and prelaminar optic nerve of hereditary optic neuropathy mouse models

**DOI:** 10.1093/braincomms/fcae404

**Published:** 2024-11-13

**Authors:** Jacques Bureau, Florence Manero, Olivier Baris, Alexia Bodin, Christophe Verny, Arnaud Chevrollier, Guy Lenaers, Philippe Codron

**Affiliations:** Laboratoire de neurobiologie et neuropathologie, Centre Hospitalier Universitaire d’Angers, 49933 Angers, France; University of Angers, Equipe MitoLab, Unité MitoVasc, INSERM U1083, CNRS 6015, SFR ICAT, 49933 Angers, France; University of Angers, SCIAM Microscopy Core Facility, SFR ICAT, F-49000, 49933 Angers, France; University of Angers, Equipe MitoLab, Unité MitoVasc, INSERM U1083, CNRS 6015, SFR ICAT, 49933 Angers, France; University of Angers, Equipe MitoLab, Unité MitoVasc, INSERM U1083, CNRS 6015, SFR ICAT, 49933 Angers, France; University of Angers, Equipe MitoLab, Unité MitoVasc, INSERM U1083, CNRS 6015, SFR ICAT, 49933 Angers, France; Service de neurologie, centre de référence des maladies neurogénétiques, Centre Hospitalier Universitaire d’Angers, 49933 Angers, France; University of Angers, Equipe MitoLab, Unité MitoVasc, INSERM U1083, CNRS 6015, SFR ICAT, 49933 Angers, France; University of Angers, Equipe MitoLab, Unité MitoVasc, INSERM U1083, CNRS 6015, SFR ICAT, 49933 Angers, France; Service de neurologie, centre de référence des maladies neurogénétiques, Centre Hospitalier Universitaire d’Angers, 49933 Angers, France; Laboratoire de neurobiologie et neuropathologie, Centre Hospitalier Universitaire d’Angers, 49933 Angers, France; University of Angers, Equipe MitoLab, Unité MitoVasc, INSERM U1083, CNRS 6015, SFR ICAT, 49933 Angers, France; Service de neurologie, centre de référence des maladies neurogénétiques, Centre Hospitalier Universitaire d’Angers, 49933 Angers, France

**Keywords:** hereditary optic neuropathy, Leber hereditary optic neuropathy, dominant optic atrophy, mitochondria, Opa1

## Abstract

Hereditary optic neuropathies, including dominant optic atrophy and Leber’s hereditary optic neuropathy, are genetic disorders characterized by retinal ganglion cell degeneration leading to vision loss, mainly associated with mitochondrial dysfunction. In this study, we analysed mitochondrial distribution and ultrastructure in the retina and longitudinal optic nerve sections of pre-symptomatic hereditary optic neuropathies mouse models with Opa1 and Nd6 deficiency to identify early mitochondrial changes. Our results show significant mitochondrial fragmentation and increased mitophagy in *Opa1^+/−^* mice, indicating early mitochondrial changes prior to neuronal loss. Conversely, *Nd6^P25L^* mice exhibited mitochondrial hypertrophy, suggesting an adaptive response to compensate for altered energy metabolism. These pre-symptomatic mitochondrial changes were mainly observed in the unmyelinated portion of the retinal ganglion cell axons, where the transmission of the visual information requires high energy expenditure, constituting the specific point of vulnerability in hereditary optic neuropathies. These findings highlight early focal mitochondrial changes prior to neuronal loss in hereditary optic neuropathies and provide insight into pre-symptomatic therapeutic approaches.

## Graphical Abstract

**Graphical Abstract:**
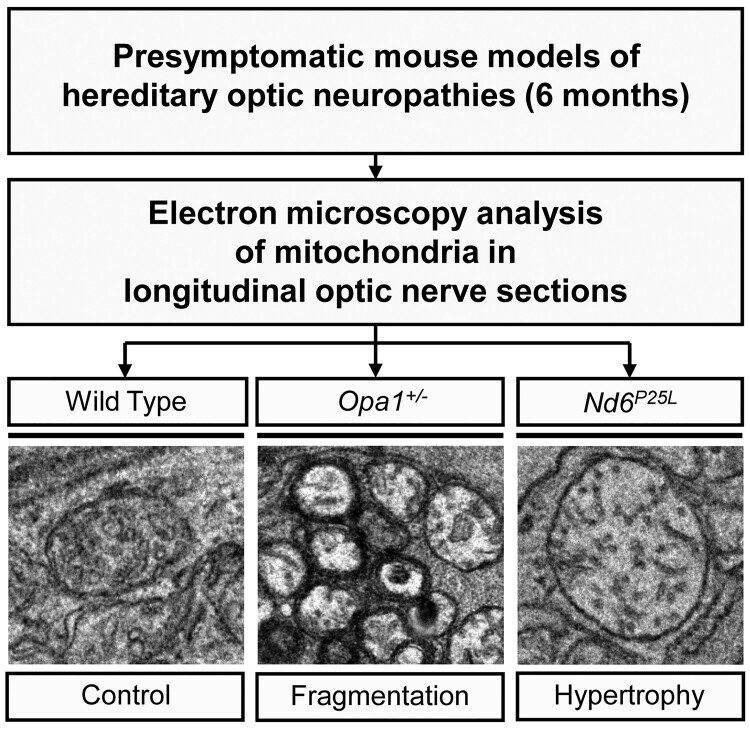

## Teaser

Bureau et al. report that early mitochondrial changes, including fragmentation and increased mitophagy in *Opa1^+/−^* mice and mitochondrial hypertrophy in *Nd6^P25L^* mice, occur before neuronal loss in hereditary optic neuropathies. These changes are predominantly observed in the unmyelinated portion of retinal ganglion cell axons, suggesting potential early therapeutic targets.

## Introduction

Hereditary optic neuropathies (HON) are genetic conditions characterized by progressive or acute degeneration of the retinal ganglion cells (RGC) responsible for the loss of central vision.^1^ Most HON are related to mitochondrial dysfunction, with mainly autosomal dominant or maternal inheritance patterns corresponding to dominant optic atrophy (DOA, MIM#165500) and Leber’s hereditary optic neuropathy (LHON, MIM#535000). DOA is the most frequent form of HON, and is caused in most patients by mutations in the mitochondrial fusion gene *Opa1*.^2-4^ LHON is associated with mutations in mitochondrial DNA encoding subunits of the respiratory chain complex I, mainly the m.3460G > A, m.11778G > A and m.14484T > C mutations in the *MT-ND1*, *MT-ND4* and *MT-Nd6* genes, respectively.^2,5,6^

Since DOA and LHON are associated with mutations in ubiquitous genes, the effect on mitochondrial dynamics and oxidative phosphorylation is likely to be systemic. However, both diseases primarily affect the optic nerve (ON), resulting in severe visual impairment. Identifying the susceptibility of RGCs to such mutations in *Opa1* or mitochondrial DNA is therefore essential to a fundamental understanding of these diseases.

The peculiar structure of the ON may provide a clue.^7^ Indeed, since RGC axons are not myelinated in the eye, high energy expenditure is required to ensure optimal non-saltatory transduction of action potentials. Conversely, after crossing the *lamina cribrosa* (LC) of the ON, RGC axons become myelinated and action potentials are maintained in an energy-saving mechanism based on saltatory propagation between the nodes of Ranvier. Initial works have shown that in response to this difference in energy demand, mitochondria in RGCs are distributed differently on either side of the LC, with numerous mitochondria in the anterior part of the ON and sparse mitochondria posterior to the LC.^7-9^

In this study, we hypothesized that the high energy demand in the unmyelinated portion of RGC axons underlies their susceptibility to mutations that affect either mitochondrial dynamics in DOA or respiratory chain complex I in LHON and may induce mitochondrial changes before neuronal loss. To this end, we studied the ultrastructure of mitochondria in the retina and throughout the emergence of the ONON of pre-symptomatic HON mouse models carrying mutations in the *Opa1* or *MT-Nd6* genes to identify early mitochondrial changes preceding RGC dysfunction and degeneration.

## Materials and methods

### Mice models

*Opa1* ^+/−^ C57BL/6 mice carrying the recurrent *Opa1* c.2708_2711delTTAG mutation,^10^  *Nd6^P25L^* C57BL/6 mice carrying the *MT-Nd6* G14600A mutation^11^ and C57BL/6 wild-type (WT) control mice were housed in clear plastic boxes in the animal facility of the Service Commun d’Animalerie Hospital-Universitaire (SCAHU, Angers, France) and subjected to standard light cycles (12 h: 90 lux light, 12 h: dark). After being anesthetized with isoflurane, *Opa1*^+/−^ (*n* = 3 males and 3 females) *Nd6^P25L^* (*n* = 3 males and 3 females) and WT (*n* = 3 males and 3 females) mice were sacrificed at 5 months old by decapitation to ensure a preserved structure of the optic nerves. Mice carrying mutations were considered pre-symptomatic at 5 months based on previous seminal studies by Sarzi *et al*. and Lin *et al*.^10,11^

### Tissue preparation

Optic nerves were removed from the base of the skull, from the retrochiasmatic pathways to the eyeball and divided into two parts by cutting through the optic chiasm.^12^ One ON was fixed and frozen in isopentane/liquid nitrogen for immunofluorescence. The other ON was immersed in a 0.1 M pH 7.4 cacodylate fixative buffer containing 2% paraformaldehyde and 2.5% glutaraldehyde for electron microscopy.

### Immunofluorescence

After freezing in pre-cooled isopentane, optic nerves were cryo-sectioned at −20°C into 20-µm-thick sections and mounted on 22 × 22 mm^2^ No. 1 coverslips. The sections were then fixed in 4% paraformaldehyde, blocked and permeabilized in 0.1% Triton X-100 and 5% bovine serum albumin and incubated overnight at 4°C with appropriate primary antibodies diluted in blocking solution. The next day, sections were washed and incubated with Alexa Fluor-conjugated secondary antibodies diluted in blocking solution for 1 h 30 min at room temperature, counterstained with Hoechst for 5 min, and washed three times in phosphate-buffered saline. Acquisitions were performed using a Zeiss Axioscop40 microscope equipped with an Axio-cam MRC5 camera, using AxioVisio 4.6 software (Carl Zeiss, Oberkochen, Germany). References and dilutions of antibodies and reagents are listed in Supplementary Table 1.

### Electron microscopy

Optic nerves were post-fixed in 1% osmium tetroxide and 1.5% potassium ferrocyanide for 2 h at room temperature, and dehydrated in a graded series of ethanol concentrations, with an intermediate stain in 3% uranyl acetate in ethanol 70° for 1 h at 4°C. After two baths in propylene oxide and one overnight bath containing both propylene oxide and EPON resin, tissues were embedded in EPON resin. Longitudinal sections were cut with an ultramicrotome UC7 (Leica Microsystems, Wetzlar, Germany) at 1 µm thickness for methylene blue + Azure blue II staining and 60 nm thickness for electron microscopy. Contrast staining was performed with 3% uranyl acetate in 50°C ethanol for 15 min, rinsed on three drops of water, then with lead citrate for 3 min, and finally rinsed on three drops of water. Samples were analysed with a JEOL JEM 1400 (JEOL, Tokyo, Japan) at 120 kV, at a 15.000× magnification, and photographed with a GATAN ORIUS 832 camera. All samples were imaged in a standardized manner. The ON was divided into four zones: (i) retina, (ii) prelaminar ON (prior to the LC), (LC) LC (junction of the unmyelinated and myelinated fibres), and (iii) retrolaminar (post-LC). A minimum of ten images were taken in each zone at 15 000× magnification. Mitochondrial density, area and circularity were measured manually in all images using ImageJ software.^13^

### Statistical analysis

Statistical analyses were performed using PRISM software version 8.0 for Windows (GraphPad, La Jolla, CA, USA). Comparisons of means were performed using Student's *t*-test for two-group analysis and one-way ANOVA for multiple-group analysis, after testing the normal distribution and homoscedasticity of the data using the Shapiro-Wilk test. In the absence of a normal distribution, nonparametric Mann–Whitney U-test and Kruskal–Wallis H-test with *post hoc* Dunn’s multiple comparison tests were used. Statistical significance thresholds were determined at *P < 0.05, **P < 0.01 and ***P < 0.001 values.

### Study approval

All protocols carried out on animals were approved by the Animal Care and Use Committee Ministry of Higher Education, Research and Innovation and recorded under the reference DUO #10627. All efforts were made to minimize the number of used animals and their suffering, according to the European directive 2010/63/UE.

## Results

### Mitochondrial enrichment in the retina and prelaminar ON in physiological conditions

**Figure 1:**
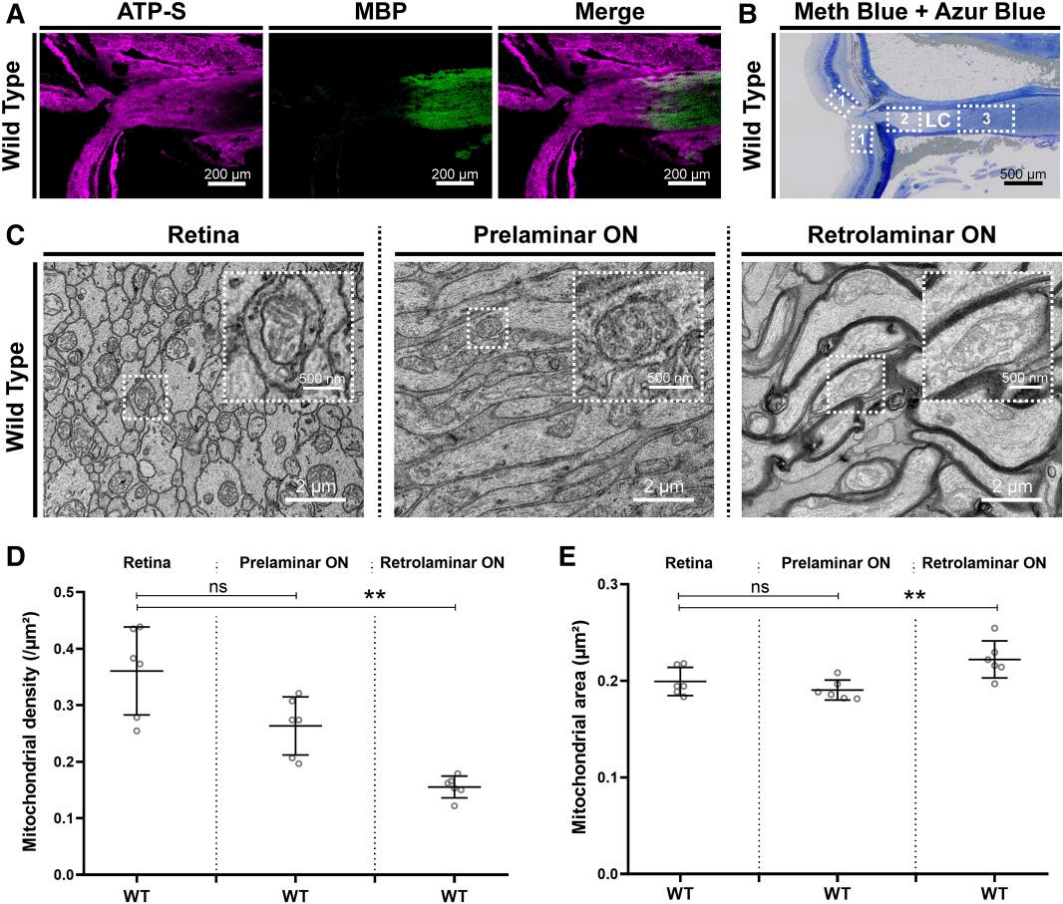
**Mitochondrial enrichment in the retina and prelaminar ON in physiological conditions.** (**A**) ON of a WT mouse immunostained for ATP-S (mitochondrial marker) and myelin basic protein (MBP, myelin marker). Immunofluorescence imaging reveals a mitochondrial enrichment at the anterior part of the ON, prior to the LC. (**B**) ON of a WT mouse stained with methylene blue and Azure blue II. All TEM images were acquired in a standardized manner, with four anatomical zones defined: 1, retina, 2, prelaminar ON, LC and 3, retrolaminar. (**C**) Representative TEM images acquired in the different parts of the ON nerve of WT mice. (**D**) Mean mitochondrial density (mitochondria/μm^2^) in RGCs at the different parts of each WT (*n* = 6) mouse. Error bars indicate mean with standard deviation. Overall comparison by Kruskal–Wallis H-test: *P* < 0.001. *Post hoc* Dunn’s multiple comparison test: ns, not significant, **P < 0.01. (**E**) Mean mitochondrial area (μm^2^) in RGCs at the different parts of the ON of each WT (*n* = 6) mouse. Error bars indicate mean with standard deviation. Overall comparison by Kruskal–Wallis H-test: *P* = 0.006. *Post hoc* Dunn’s multiple comparison test: ns, not significant, **P < 0.01.

None of the mice in the three study groups showed any visual, neurological or general symptoms during the study, in agreement with their initial description.^10,11^ Mice were sacrificed at 5 months for histologic examination of the optic nerves. In total, 900 images were acquired, and 21.894 mitochondria were analysed. As previously reported, higher mitochondrial density was observed by immunofluorescence microscopy in the prelaminar ON compared to the retrolaminar ON in WT mice, where almost no mitochondrial staining was seen (Fig. 1A). Similarly, transmission electron microscopy (TEM) analysis showed that mitochondrial density (mitochondria/µm^2^) was 2-fold higher in the pre-LC zone compared to the retrolaminar ON (*P* < 0.001, Fig. 1B–D). Mitochondria in the retina and in the pre-LC zone also had a smaller area (µm^2^) compared to those in the retrolaminar ON (*P* = 0.006) (Fig. 1C and E). Taken together, these results demonstrate that mitochondrial morphology and density differ between the unmyelinated and myelinated portions of the RGC under physiological conditions, supporting distinct dynamics and energy metabolism depending on the action potential transmission mode.

### Mitochondrial fragmentation, increased mitophagy and protein aggregation in the retina and prelaminar ON of pre-symptomatic *Opa1*^+/−^ mice

**Figure 2:**
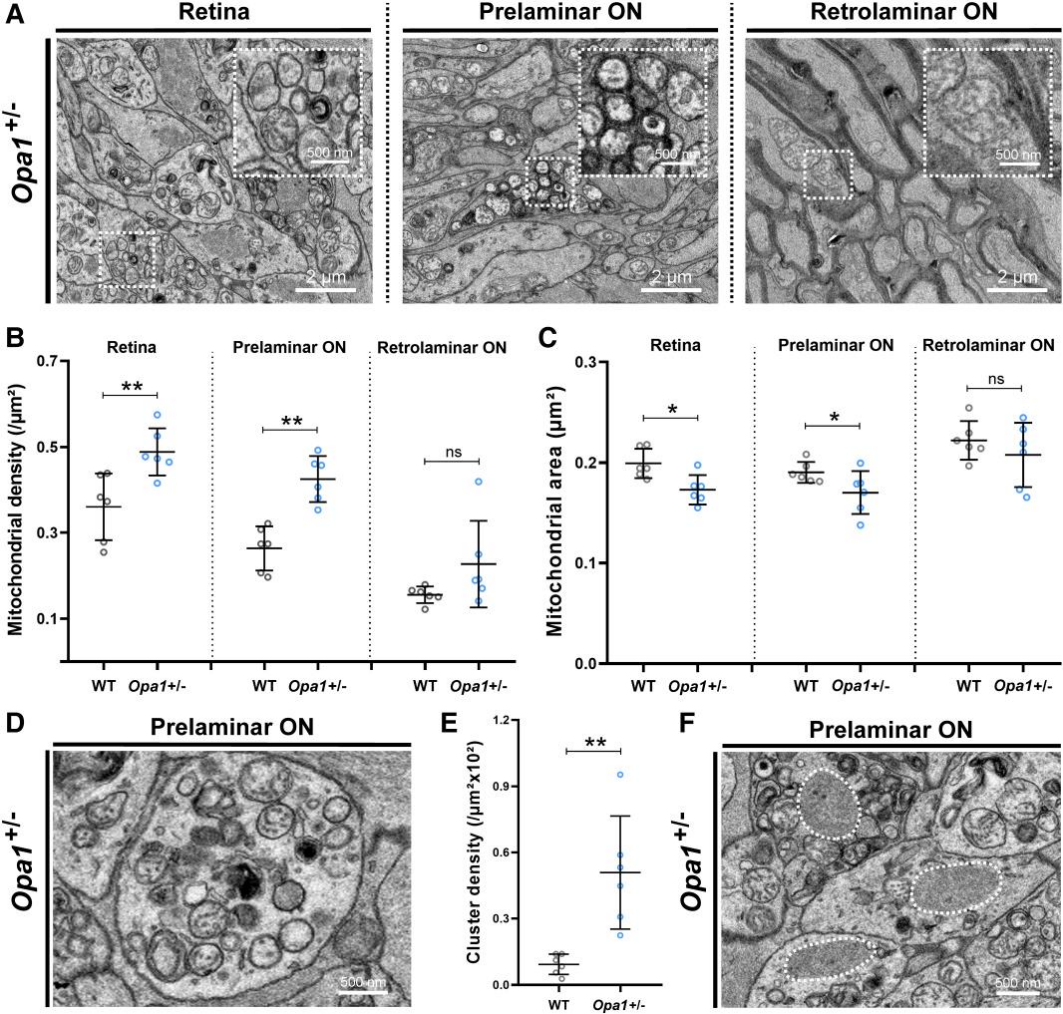
**Mitochondrial fragmentation, increased mitophagy and protein aggregation in the retina and prelaminar ON of pre-symptomatic *Opa1*^+/−^ mice.** (**A**) Representative TEM images acquired in the different parts of the ON nerve of *Opa1*^+/−^ mice. (**B**) Mean mitochondrial density (mitochondria/μm^2^) in RGCs at the different parts of the ON of each *Opa1*^+/−^ (*n* = 6) and WT (*n* = 6) mouse. Error bars indicate mean with standard deviation. Pairwise comparison with Mann–Whitney U-test: ns, not significant, **P < 0.01. (**C**) Mean mitochondrial area (μm^2^) in RGCs at the different parts of the ON of each *Opa1*^+/−^ (*n* = 6) and WT (*n* = 6) mouse. Error bars indicate mean with standard deviation. Pairwise comparison with Mann–Whitney U-test: ns, not significant, *P < 0.05. (**D**) Representative TEM image of mitophagy clusters observed in RGCs in the retina and prelaminar ON of *Opa1*^+/−^ mice. (**E**) Mean cluster density (/μm^2^ × 10^2^) in the different prelaminar ON of each *Opa1*^+/−^ (*n* = 6) and WT (*n* = 6) mouse. Error bars indicate mean with standard deviation. Pairwise comparison with Mann–Whitney U-test: **P < 0.01. (**F**) Representative TEM image of proteinaceous aggregates observed in the prelaminar ON of *Opa1*^+/−^ mice (surrounded by dotted lines).

We then analysed the density and morphology of mitochondria on both sides of the LC in *Opa1*^+/−^ mice carrying the most frequent *Opa1* mutation found in human DOA patients.^10^ In the retina and the prelaminar ON of *Opa1*^+/−^ mice, a major phenotype was already visible within axons with more mitochondria (/µm^2^, *P* = 0.002) of smaller area (*P* = 0.026) compared to WT mice (Fig. 2A–C), suggesting a fragmentation of the mitochondrial network. These features were also observed in the retrolaminar ON although not significantly (*P* = 0.649 and *P* = 0.589, respectively). In addition, TEM images revealed numerous clusters of mitophagy in enlarged axons in the retina and prelaminar ON of *Opa1*^+/−^ mice (Fig. 2D). The density of these prelaminar clusters was significantly higher in *Opa1*^+/−^ mice compared to WT mice (*P* = 0.002, Fig. 2E). No cluster was observed in the retrolaminar ON of the *Opa1*^+/−^ mice. Finally, multiple protein aggregates were seen in the retina and prelaminar ON of *Opa1*^+/−^ mice, which were not seen in the other genotypes (Fig. 2F). These data indicate that the *Opa1* c.2708_2711delTTAG mutation causes pre-symptomatic mitochondrial changes in the anterior part of the ON, which are indicative of mitochondrial fragmentation, increased mitophagy and axonal transport defects.

### Mitochondrial hypertrophy in the retina and throughout the optic nerve of pre-symptomatic *Nd6^P25L^* mice

**Figure 3:**
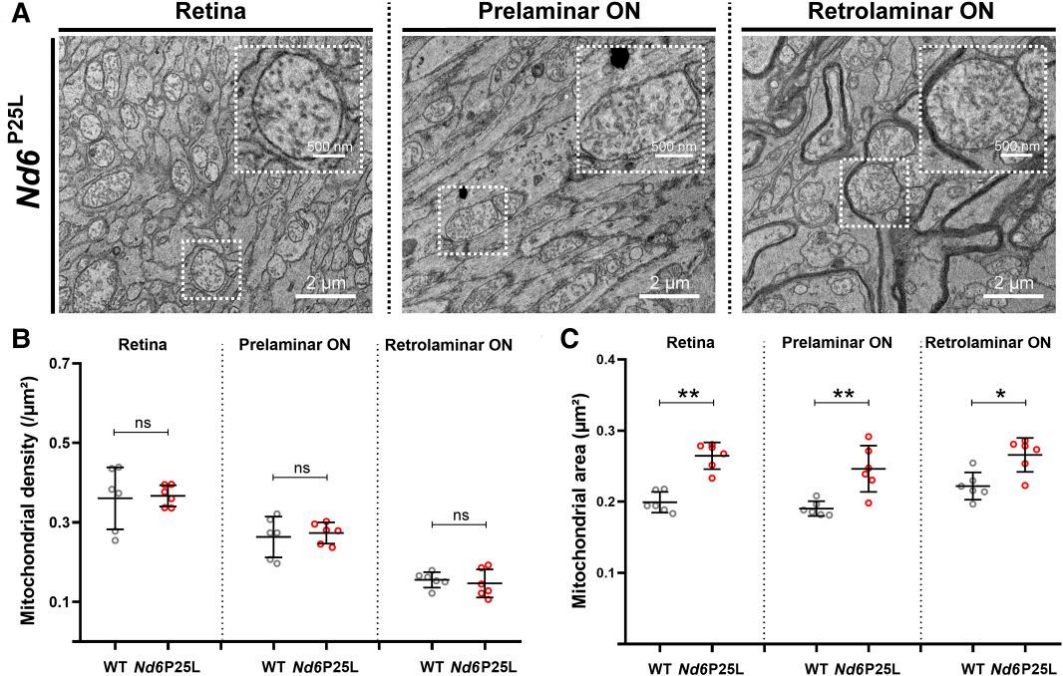
**Mitochondrial hypertrophy in the retina and throughout the ON of pre-symptomatic Nd6^P25L^ mice.** (**A**) Representative TEM images acquired in the different parts of the ON nerve of *Nd6^P25L^* mice. (**B**) Mean mitochondrial density (mitochondria/μm^2^) in RGCs at the different parts of the ON of each *Nd6^P25L^* (*n* = 6) and WT (*n* = 6) mouse. Error bars indicate mean with standard deviation. Pairwise comparison with Mann–Whitney U-test: ns, not significant. (**C**) Mean mitochondrial area (μm^2^) in RGCs at the different parts of the ON of each *Nd6^P25L^* (*n* = 6) and WT (*n* = 6) mouse. Error bars indicate mean with standard deviation. Pairwise comparison with Mann–Whitney U-test: *P < 0.05, **P < 0.01.

We next focused on the density and morphology of mitochondria in *Nd6^P25L^* mice. No difference in mitochondrial density (mitochondria/µm^2^) was observed between *Nd6^P25L^* and WT mice in the retina and pre- and post-LC areas (*P* = 0.937, *P* = 0.783 and *P* = 0.621 respectively, Fig. 3A and B). However, mitochondria had a significantly larger area in *Nd6^P25L^* mice compared to WT mice, on both sides of the LC, but more pronounced in the retina and the prelaminar ON (*P* = 0.002 and *P* = 0.004, respectively, Fig. 3A and C). In contrast to *Opa1*^+/−^ mice, no mitophagy cluster or protein aggregate was observed in the optic nerves of *Nd6^P25L^* mice. These data indicate that the *MT-Nd6* mutation causes pre-symptomatic mitochondrial changes indicative of mitochondrial biogenesis or enhanced mitochondrial fusion, which are more pronounced in the anterior part of the ON.

### Absence of mitochondrial alteration in astrocytes in the optic nerves of *Opa1*^+/−^ and *Nd6^P25L^* mice

Mitochondria from astrocytes were normal all along the ON regardless of the mouse genotype. In addition, astrocytic mitochondria differed from those of axons in having a higher density of cristae (Supplementary Fig. 1). These observations indicate that pre-symptomatic mitochondrial alterations are specific to pre-LC region of RGC in *Opa1*^+/−^ and *Nd6^P25L^* mice.

## Discussion

In this study, we demonstrate early mitochondrial changes in the retina and the prelaminar ON of mouse models of hereditary optic atrophy carrying mutations in the *Opa1* and *MT-Nd6* genes. We first examined mitochondria longitudinally along RGC axons from the retina to the retrolaminar ON in WT mice. As previously reported, we observed a higher mitochondrial density in the retina and prelaminar ON, supporting the hypothesis that these unmyelinated sites require more mitochondrial oxidative phosphorylation and ATP to transmit visual information in a non-saltatory mode, constituting a specific point of vulnerability in HON. Based on this observation, we hypothesized that mutations affecting mitochondrial dynamics or metabolism result in early visible changes in the ON.

We first examined the retina and ON of pre-symptomatic mice carrying the *Opa1* c.2708_2711delTTAG mutation responsible for DOA in patients.^10^ Opa1 is a mitochondrial protein that, together with Mfn1 and Mfn2, participates in mitochondrial fusion and maintains the integrity of the mitochondrial network.^14,15^ Haplo insufficiency has been proposed as the predominant disease mechanism underlying *Opa1*-related DOA, as the majority of disease-causing variants result in premature translation termination.^16^ Downregulation of *Opa1* in cells leads to fragmentation of the mitochondrial network with increased mitophagy.^17^ Interestingly, these features were observed in the retina and the anterior part of the ON in *Opa1*^+/−^ mice at an early stage, before initiation of neuronal loss. Although located in a different prelaminar RGC region, our data parallel previous observations showing increased mitophagy and autophagosome accumulation at the RGC hillock associated with local activation of the AMPK–ULK1 axis.^18^ We also observed protein aggregates within prelaminar RGC fibres, which could represent markers of axonal transport defects due to altered energy metabolism and/or mechanical congestion by mitophagy clusters.^19^ This is an additional and, to our knowledge, undescribed mechanism of neuronal toxicity that may contribute specifically to neurodegeneration in HON diseases, especially since small-calibre axons show a higher vulnerability to *Opa1* mutation in an aged mouse model of DOA.^20^ The nature of these proteins observed aggregated in the ON is important to define in future studies to better understand their origin and pathogenesis in DOA.

Examination of the ON in *Nd6^P25L^* mice also revealed early mitochondrial changes, strikingly, different from those found in *Opa1*^+/−^ mice. Mitochondria were enlarged throughout the ON, but more so in the retina and prelaminar ON. This hypertrophy, which has been reported in tissues from LHON mutation carriers,^21^ may reflect increased mitochondrial biogenesis and metabolism to compensate for altered energy production due to the mutations of complex I subunits. We can also hypothesize that transport of hypertrophic mitochondria might be more compromised in small-calibre axons than in larger ones, explaining why the former are more susceptible to dysfunction and degeneration than the latter. Our data are somehow divergent with previous studies that disclosed increased mitophagy in LHON cell lines, including induced pluripotent stem cells-derived neurons,^22^ suggesting that according to the cell type and environmental conditions, LHON mutations can display divergent mitochondrial and cellular phenotypes, although converging to a balance between mitochondrial degradation and biosynthesis. In any case, this mitochondrial adaptive response could maintain unaffected mutation carriers until compensatory mechanisms are overcome, explaining the incomplete penetrance of LHON and the rapid visual impairment observed in the disease.^23,24^ No mitophagy clusters or protein aggregates were observed, conversely to what was disclosed in *Opa1*^+/−^ mice.

The early mitochondrial changes observed in the unmyelinated parts of RGC in mouse models of hereditary optic atrophy are reported here for the first time. Indeed, previous ultrastructural analyses of the ON in mouse models with impaired Opa1, Nd4 or Nd6 function were performed on the post-laminar myelinated portion of the ON, with transverse sections, and at late time points after significant axonal loss.^10,11,25-30^ The early and pronounced mitochondrial changes observed in our study in a site of high energy demand indicate a pre-symptomatic zone of increased fragility in HON. These findings underscore the potential for targeted mitochondrial therapies in the early stages of HON and suggest that local treatment could pre-emptively mitigate neuronal loss. Furthermore, the distinct mitochondrial alterations associated with *Opa1* and *MT-Nd6* mutations highlight the importance of personalized therapeutic approaches, tailored to the specific mitochondrial dysfunction underlying different forms of HONs. Finally, our results provide evaluation criteria for pre-symptomatic treatments under experimental conditions.

## Supplementary Material

fcae404_Supplementary_Data

## Data Availability

The data that support the findings of this study are available from the corresponding author upon reasonable request.

## References

[1] Yu-Wai-Man P, Griffiths PG, Hudson G, Chinnery PF. Inherited mitochondrial optic neuropathies. J Med Genet. 2009;46:145–158. 19001017 10.1136/jmg.2007.054270 PMC2643051

[2] Rocatcher A, Desquiret-Dumas V, Charif M, et al The top 10 most frequently involved genes in hereditary optic neuropathies in 2186 probands. Brain. 2023;146:455–460. 36317462 10.1093/brain/awac395

[3] Alexander C, Votruba M, Pesch UE, et al OPA1, encoding a dynamin-related GTPase, is mutated in autosomal dominant optic atrophy linked to chromosome 3q28. Nat Genet. 2000;26:211–215. 11017080 10.1038/79944

[4] Delettre C, Lenaers G, Griffoin JM, et al Nuclear gene OPA1, encoding a mitochondrial dynamin-related protein, is mutated in dominant optic atrophy. Nat Genet. 2000;26:207–110. 11017079 10.1038/79936

[5] Wallace DC, Singh G, Lott MT, et al Mitochondrial DNA mutation associated with Leber’s hereditary optic neuropathy. Science. 1988;242:1427–1430. 3201231 10.1126/science.3201231

[6] Mackey DA, Oostra RJ, Rosenberg T, et al Primary pathogenic mtDNA mutations in multigeneration pedigrees with Leber hereditary optic neuropathy. Am J Hum Genet. 1996;59:481–485. 8755941 PMC1914749

[7] Lenaers G, Neutzner A, Le Dantec Y, et al Dominant optic atrophy: Culprit mitochondria in the optic nerve. Prog Retin Eye Res. 2021;83:100935. 33340656 10.1016/j.preteyeres.2020.100935

[8] Bristow EA, Griffiths PG, Andrews RM, et al The distribution of mitochondrial activity in relation to optic nerve structure. Arch Ophthalmol. 2002;120:791–796. 12049585 10.1001/archopht.120.6.791

[9] Barron MJ, Griffiths P, Turnbull DM, et al The distributions of mitochondria and sodium channels reflect the specific energy requirements and conduction properties of the human optic nerve head. Br J Ophthalmol. 2004;88:286–290. 14736793 10.1136/bjo.2003.027664 PMC1771975

[10] Sarzi E, Angebault C, Seveno M, et al The human OPA1delTTAG mutation induces premature age-related systemic neurodegeneration in mouse. Brain. 2012;135:3599–3613. 23250881 10.1093/brain/aws303

[11] Lin CS, Sharpley MS, Fan W, et al Mouse mtDNA mutant model of Leber hereditary optic neuropathy. Proc Natl Acad Sci U S A. 2012;109:20065–20070. 23129651 10.1073/pnas.1217113109 PMC3523873

[12] Pozyuchenko K, Shouchane-Blum K, Brody J, et al Investigating animal models of optic neuropathy: An accurate method for optic nerve and chiasm dissection in mice. J Neurosci Methods. 2020;331:108527. 31775012 10.1016/j.jneumeth.2019.108527

[13] Schindelin J, Arganda-Carreras I, Frise E, et al Fiji: An open-source platform for biological-image analysis. Nat Methods. 2012;9:676-–6682. 22743772 10.1038/nmeth.2019 PMC3855844

[14] Cipolat S, Martins de Brito O, Dal Zilio B, Scorrano L. OPA1 requires mitofusin 1 to promote mitochondrial fusion. Proc Natl Acad Sci U S A. 2004;101:15927–15932. 15509649 10.1073/pnas.0407043101 PMC528769

[15] Chen H, Detmer SA, Ewald AJ, et al Mitofusins Mfn1 and Mfn2 coordinately regulate mitochondrial fusion and are essential for embryonic development. J Cell Biol. 2003;160:189–200. 12527753 10.1083/jcb.200211046 PMC2172648

[16] Le Roux B, Lenaers G, Zanlonghi X, et al OPA1: 516 unique variants and 831 patients registered in an updated centralized Variome database. Orphanet J Rare Dis. 2019;14:214. 31500643 10.1186/s13023-019-1187-1 PMC6734442

[17] Kane MS, Alban J, Desquiret-Dumas V, et al Autophagy controls the pathogenicity of OPA1 mutations in dominant optic atrophy. J Cell Mol Med. 2017;21:2284–2297. 28378518 10.1111/jcmm.13149 PMC5618673

[18] Zaninello M, Palikaras K, Naon D, et al Inhibition of autophagy curtails visual loss in a model of autosomal dominant optic atrophy. Nat Commun. 2020;11:4029. 32788597 10.1038/s41467-020-17821-1 PMC7423926

[19] Okan ICT, Ozdemir F, Agca C. Axonal transport defects in retinal ganglion cell diseases. Adv Exp Med Biol. 2023;1415:223–227. 37440037 10.1007/978-3-031-27681-1_32

[20] González-Menéndez I, Reinhard K, Tolivia J, et al Influence of Opa1 mutation on survival and function of retinal ganglion cells. Invest Ophthalmol Vis Sci. 2015;56:4835–4845. 26218912 10.1167/iovs.15-16743

[21] Giordano C, Iommarini L, Giordano L, et al Efficient mitochondrial biogenesis drives incomplete penetrance in Leber’s hereditary optic neuropathy. Brain. 2014;137:335–353. 24369379 10.1093/brain/awt343 PMC3914475

[22] Danese A, Patergnani S, Maresca A, et al Pathological mitophagy disrupts mitochondrial homeostasis in Leber’s hereditary optic neuropathy. Cell Rep. 2022;40:111124. 35858578 10.1016/j.celrep.2022.111124 PMC9314546

[23] Kerrison JB, Newman NJ. Clinical spectrum of Leber’s hereditary optic neuropathy. Clin Neurosci. 1997;4:295–301. 9292259

[24] Yu-Wai-Man P, Chinnery P, Adam M, et al Leber hereditary optic neuropathy. GeneReviews. 2021. Accessed 25 November 2024. https://www.ncbi.nlm.nih.gov/books/NBK1174/

[25] Alavi MV, Bette S, Schimpf S, et al A splice site mutation in the murine Opa1 gene features pathology of autosomal dominant optic atrophy. Brain. 2007;130:1029–1042. 17314202 10.1093/brain/awm005

[26] Davies VJ, Hollins AJ, Piechota MJ, et al Opa1 deficiency in a mouse model of autosomal dominant optic atrophy impairs mitochondrial morphology, optic nerve structure and visual function. Hum Mol Genet. 2007;16:1307–1318. 17428816 10.1093/hmg/ddm079

[27] White KE, Davies VJ, Hogan VE, et al OPA1 deficiency associated with increased autophagy in retinal ganglion cells in a murine model of dominant optic atrophy. Invest Ophthalmol Vis Sci. 2009;50:2567–2571. 19234344 10.1167/iovs.08-2913

[28] Williams PA, Piechota M, von Ruhland C, et al Opa1 is essential for retinal ganglion cell synaptic architecture and connectivity. Brain. 2012;135:493–505. 22300878 10.1093/brain/awr330

[29] Yu H, Ozdemir SS, Koilkonda RD, et al Mutant NADH dehydrogenase subunit 4 gene delivery to mitochondria by targeting sequence-modified adeno-associated virus induces visual loss and optic atrophy in mice. Mol Vis. 2012;18:1668–1683. 22773905 PMC3388991

[30] Yu H, Koilkonda RD, Chou TH, et al Consequences of zygote injection and germline transfer of mutant human mitochondrial DNA in mice. Proc Natl Acad Sci U S A. 2015;112:E5689–E5698. 26438859 10.1073/pnas.1506129112 PMC4620890

