# Supplementary material for: Opa1 and MT-Nd6 mutations induce early mitochondrial changes in the retina and prelaminar optic nerve of hereditary optic neuropathy mouse models
Source: Brain Commun. 2024 Nov 13;6(6):fcae404. doi: 10.1093/braincomms/fcae404 (PMC11630736; doi:10.1093/braincomms/fcae404)
Supplement: fcae404_Supplementary_Data [file fcae404_supplementary_data.pdf]

Supplementary Table 1: Antibodies and reagents.

Primary antibodies	Host	Company	Catalog Number	Dilution
ATP synthase (subunit β)	Mouse	Thermo Fisher Scientific	A21351	1:1000
Myelin Basic Protein	Rabbit	Dako-Agilent	A0623	1:250
**Secondary antibodies**	**Host**	**Company**	**Catalog Number**	**Dilution**
Alexa Fluor 488 anti-Rabbit IgG	Goat	Thermo Fisher Scientific	A-11008	1:3000
Alexa Fluor 568 anti-Mouse IgG	Goat	Thermo Fisher Scientific	A-11004	1:3000

Reagents or Resources	Company	Catalog Number
Bovine Serum Albumin	Sigma-Aldrich	A6003
Hoechst 33342	Thermo Fisher Scientific	H3570
Microscope cover glasses	Paul Marienfeld	0102052
Paraformaldehyde	Sigma-Aldrich	P6148
Phosphate Buffered Saline	Pan Biotech	P04-53500
Triton X-100	Sigma-Aldrich	T9284

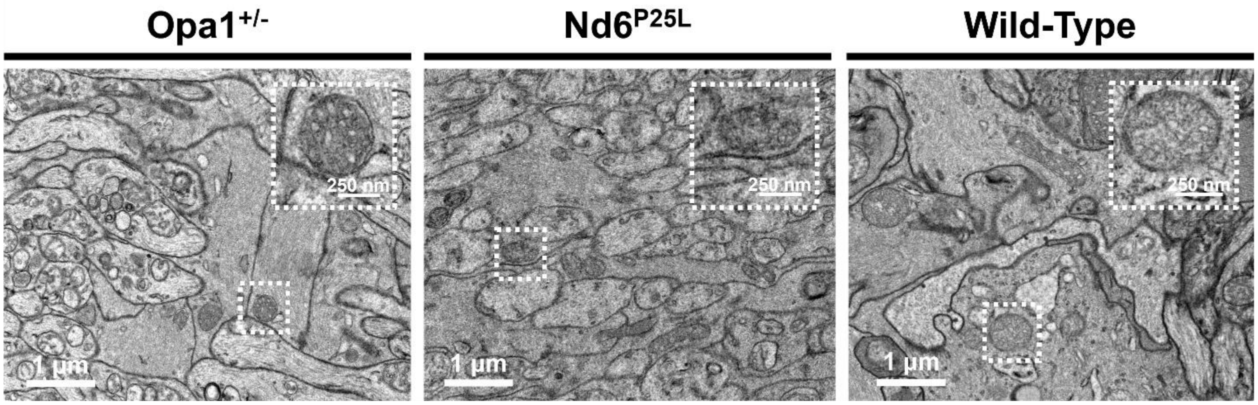

Supplementary Figure 1: Representative images of mitochondria from astrocytes in the optic nerve of *Opa1*$^{+/-}$, *Nd6*^{P25L} and WT mice.
